# “*Every method seems to have its problems”-* Perspectives on side effects of hormonal contraceptives in Morogoro Region, Tanzania

**DOI:** 10.1186/s12905-015-0255-5

**Published:** 2015-11-03

**Authors:** Joy J. Chebet, Shannon A. McMahon, Jesse A. Greenspan, Idda H. Mosha, Jennifer A. Callaghan-Koru, Japhet Killewo, Abdullah H. Baqui, Peter J. Winch

**Affiliations:** Department of International Health, Johns Hopkins Bloomberg School of Public Health, 615 N. Wolfe Street, Baltimore, MD USA; Institute of Public Health, Ruprecht-Karls-Universität, Im Neuenheimer Feld 324, 69120 Heidelberg, Germany; Department of Behavioural Sciences, Muhimbili University of Health and Allied Sciences, P.O. Box 65015, Dar es Salaam, Tanzania; Department of Epidemiology and Biostatistics, Muhimbili University of Health and Allied Sciences, P.O. Box 65015, Dar es Salaam, Tanzania

**Keywords:** Hormonal contraception, Contraceptive side effects, Family planning, Postpartum women, Tanzania

## Abstract

**Background:**

Family planning has been shown to be an effective intervention for promoting maternal, newborn and child health. Despite family planning's multiple benefits, women's experiences of - or concerns related to - side effects present a formidable barrier to the sustained use of contraceptives, particularly in the postpartum period. This paper presents perspectives of postpartum, rural, Tanzanian women, their partners, public opinion leaders and community and health facility providers related to side effects associated with contraceptive use.

**Methods:**

Qualitative interviews were conducted with postpartum women (*n* = 34), their partners (*n* = 23), community leaders (*n* = 12) and health providers based in both facilities (*n* = 12) and communities (*n* = 19) across Morogoro Region, Tanzania. Following data collection, digitally recorded data were transcribed, translated and coded using thematic analysis.

**Results:**

Respondents described family planning positively due to the health and economic benefits associated with limiting and spacing births. However, side effects were consistently cited as a reason that women and their partners choose to forgo family planning altogether, discontinue methods, switch methods or use methods in an intermittent (and ineffective) manner. Respondents detailed side effects including excessive menstrual bleeding, missed menses, weight gain and fatigue. Women, their partners and community leaders also described concerns that contraceptives could induce sterility in women, or harm breastfeeding children via contamination of breast milk. Use of family planning during the postpartum period was viewed as particularly detrimental to a newborn’s health in the first months of life.

**Conclusions:**

To meet Tanzania’s national target of increasing contraceptive use from 34 to 60 % by 2015, appropriate counseling and dialogue on contraceptive side effects that speaks to pressing concerns outlined by women, their partners, communities and service providers are needed.

## Background

Modern contraception has been widely promoted as a mechanism to improve health, avert maternal and child death, and stabilize population growth [1–3]. Nevertheless, many countries and regions—particularly in sub-Saharan Africa (SSA)—lag in the adoption of modern contraception [1]. The postpartum period (PPP), which spans from delivery to six weeks and continues for one year after birth in the extended PPP [4], is considered a “critical time” to reach women (and their families) and provide key information regarding family planning (FP) including: counseling on healthy timing and spacing of pregnancy; return to fertility and pregnancy risk after childbirth; appropriate contraceptive options; the lactational amenorrhea method (LAM) and timely transition to another modern method [5]. At present, the level of unmet need during this period is high and women in the PPP have been described as an “obvious… but often overlooked” audience for FP interventions and messaging [5].

Research explicitly focused on the FP experience of postpartum women has expanded since the 2012 London Summit on FP, which bolstered a renewed international emphasis on FP and highlighted the potential for postpartum family planning (PPFP) to accelerate progress toward Millennium Development Goals 4 and 5. This commitment was reinforced at the 2013 International Conference on Family Planning in Addis Ababa, Ethiopia where a gathering of technical experts drafted the “Programming Strategies for Postpartum Family Planning” to serve as a reference for those engaged in PPFP, with an emphasis on low- and middle-income settings [5].

Following these two galvanizing meetings, at least two studies across multiple, low-income settings have found that pregnancies are not optimally timed, and women experience an unmet need for FP during the PPP [6, 7]. Moore’s 2015 study across 21 low- and middle-income countries found that unmet need had not changed demonstrably since a seminal, 2001 analysis [8] and 61 % of postpartum women across all countries included in the study have an unmet need for family planning [6].

Interventions and research to understand the needs of postpartum women have been described as “urgently needed” in order to “reduce unmet need and to improve both maternal and infant outcomes, especially amongst young women” [7]. Research has expanded in SSA with studies conducted in Burkina Faso [9], Ghana [10], Kenya [11] and Ethiopia [12]. Unifying themes across these studies include: the need to strengthen FP counseling, access and uptake in order to address unmet FP needs. Unfortunately, to date “very little is known about how pregnant women in SSA arrive at their PPFP decisions” [10].

This paper examines the knowledge of, attitudes toward and experiences with FP-- with a particular emphasis on contraceptive side effects from hormonal contraceptives (pills, injectables and implants)-- as described by respondents based in communities and facilities across the Morogoro Region of Tanzania.

### Study context

#### Tanzania

Tanzania has a population of 43 million [13], 25 % of whom live below the poverty line [14]. The total fertility rate (TFR) is 5.4 children per woman, with a TFR of 6.1 and 3.7 among rural and urban women, respectively [13]. Factors contributing to Tanzania’s high TFR include a relatively low median age at first marriage (18.9 years) and low median age at first birth (19.5 years) among women ages 20–49 [13]. Another contributor to high fertility is the country’s low contraceptive prevalence rate (CPR). CPR has risen steadily among married Tanzanian women, from 26 % in 2004/5 to 34.4 % in 2010, and a majority of health facilities (76 %)—including nearly all government-run facilities (97 %)—have some form of a modern contraception available [15]. Nevertheless, contraceptive use remains low with 27.4 % using modern methods, including injectables (10.6 %), pills (6.7 %), sterilization (3.5 %), male condoms (2.3 %), implants (2.3 %), LAM (1.3 %) and intrauterine devices (0.6 %) [13].

Nearly all women (97 %) attend at least one antenatal care visit during pregnancy, approximately half of women deliver in a health facility (50.2 %) and roughly a third of women (35.4 %) attend a postnatal checkup. A majority of Tanzanian adults—both men and women—can name multiple types of modern contraceptives, with oral contraceptives, male condoms, injectables and implants being the most commonly recognized methods [13]. Still, 25.3 % of married women in Tanzania have an unmet need for FP, with 15.9 % reporting an unmet need for birth spacing and 9.5 % reporting an unmet need for limiting pregnancies [13].

#### Morogoro Region

Morogoro Region was the setting for this research, which was conducted as a part of a larger program evaluation of a maternal health program implemented by the Tanzanian Ministry of Health and Social Welfare (MOHSW) in the region (details of the program have been published elsewhere) [16].

Situated in Eastern Tanzania, the region has a population of 2.2 million and a population density of 31 inhabitants per square kilometer, making it one of Tanzania’s geographically largest (70,000 sq. km) and yet least densely populated areas [17]. The unmet need for FP among women in Morogoro Region is slightly lower than in the country overall, with 22.6 % of married women reporting an unmet need (15.2 % for birth spacing and 7.4 % for birth limiting) [13]. Among reproductive age women in the region, CPR is higher (39.9 %) than the national average (23.6 %), and the most commonly used FP methods are injectables (11 %), pills (7 %) and female sterilization (4 %) [13]. The median duration of breastfeeding in the Eastern Zone (which encompasses Morogoro) is 21.6 months, and exclusive breastfeeding is undertaken for one month (compared to national averages of 20.4 and 4.1 months, respectively) [13]. The median duration of amenorrhea among women who gave birth in the three years preceding the survey in Morogoro Region is 6.1 months (9.8 months nationally) and the median duration of postpartum abstinence is 6.2 months (3.8 nationally) [13]. Some form of modern contraception is available in most facilities (60 %) [15].

## Methods

### Study design

The study design was informed by rapid ethnographic approaches developed as part of applied qualitative research for health [18–20]. Rapid ethnographic approaches, or “quick ethnography,” represents an adaptation of traditional (long term) anthropological inquiry with an aim to reduce the duration of time for fieldwork in order to provide timely insights to program planners, evaluators and stakeholders [21]. Similar to traditional ethnography, rapid ethnography subscribes to the constructivist worldview, which posits that knowledge is socially constructed [22, 23]. Because the goal of this research was to inform a program that promotes community-facility linkages, and aims to understand access to and opinions of facility-based maternal health services, the research team conducted in-depth interviews (IDIs) across a variety of respondents: postpartum women, their partners, community leaders (including religious and opinion leaders), community health workers (CHWs) and facility-based providers. These interviews explored several aspects of reproductive health including knowledge and use of FP, barriers to accessing and consistently using FP, and sources of influence that shape women’s perceptions and decisions related to FP.

### Sampling

Sampling among the community-based respondent groups was stratified by communities living near (<3 km) and far (3–10 km) from health facilities. This was done across 16 villages in the catchment areas of 8 government health centers (8 villages near, and 8 villages far from a health center) in four districts of the region (Morogoro, Mvomero, Kilosa^1^ and Ulanga District Council). Stratification was done with an intention of exploring nuances in utilization of maternal, neonatal and child health services across districts and by distance to facilities. Facility-based providers helped the research team identify distant villages by discussing with the research team communities that were situated within their catchment area but were known to seldom interact with the formal health system. Once in communities, the data collection team presented themselves to village leaders (this sometimes included CHWs) and asked to be introduced to any woman known to have delivered in the preceding 14 months who may be available for an interview in the coming days. The 14-month time period was chosen as it reduced recall bias, but allowed enough time for women to reinitiate FP. Although couples were prioritized for IDIs, roughly two-thirds of all male partners were not available to participate. Prior FP use was not a criterion for participation, although nearly all postpartum women had prior experience using FP. Interviewed leaders included religious leaders, as well as members of an elected village board and/or village health committee. These individuals also assisted the research team in identifying CHWs. Leaders and CHWs were interviewed irrespective of gender, age, education level or length of service. Leaders and CHWs helped data collectors identify women and their partners. In addition, data collectors canvassed the village and invited eligible mothers and fathers to participate. A second phase of data collection included IDIs with facility-based providers. In total, 100 IDIs were conducted (88 community-based interviews and 12 facility-based provider interviews) (See Table 1).

**Table 1:** Demographic characteristics of postpartum women, their partners, community leaders and health providers

	Postpartum women^a^ (*n* = 34)	Partners of postpartum women^b^ (*n* = 23)	Community leaders ^c^ (*n* = 12)	Community health workers (*n* = 19)	Facility based providers (*n* = 12)
Age (years)					
Mean	28.56	37.14	43.00	42.66	37.17
	(*n* = 34)	(*n* = 22)	(*n* = 11)	(*n* = 18)	(*n* = 12)
Median	28	37	42	43	35
Range	18–43	23–60	29–65	25–52	20–57
Gender					
Female	35 (100.00)	0 (0.00)	0 (0.00)	9 (47.37)	9 (75.00)
Male	0 (0.00)	23 (100.00)	12 (100.00)	10 (52.63)	3 (25.00)
District—*n* (%)					
Morogoro rural	1 (2.94)	1 (4.35)	0 (0.00)	2 (10.52)	3 (25.00)
Mvomero	12 (35.29)	5 (21.74)	4 (33.33)	7 (36.84)	0 (0.00)
Kilosa	10 (29.41)	11 (47.83)	3 (25.00)	3 (15.79)	9 (75.00)
Ulanga	11 (32.35)	6 (26.09)	5 (41.67)	7 (36.84)	0 (0.00)
Marital status-*n* (%)					
Married/Cohabiting	29 (85.29)	21 (91.30)	11 (91.67)	13 (68.42)	--
Single	5 (14.71)	1 (4.35)	0 (0.00)	3 (15.79)	--
Divorced	0 (0.00)	0 (0.00)	0 (0.00)	2 (10.53)	--
Not reported	0 (0.00)	1 (4.35)	1 (8.33)	1 (5.26)	--
Level of education					
No education	5 (14.71)	3 (13.04)	0 (0.00)	0 (0.00)	--
Started primary school	6 (17.65)	2 (8.70)	0 (0.00)	0 (0.00)	--
Completed primary school	17 (50.00)	15 (65.22)	7 (58.33)	13 (68.42)	--
Started secondary school	0 (0.00)	0 (0.00)	1(8.33)	1 (5.26)	--
Completed secondary school	3 (8.82)	2 (8.70)	1 (8.33)	2 (10.53)	--
Other	0 (0.00)	0 (0.00)	1 (8.33)	1 (5.26)	--
Not reported	3 (8.82)	1 (4.35)	2 (16.67)	2 (10.53)	--
Self reported literacy *n* (%)					
Literate	23 (67.65)	20 (86.96)	11 (91.67)	19 (100.00)	--
Illiterate	10 (29.41)	1 (4.35)	0 (0.00)	0 (0.00)	--
Not reported	1 (2.94)	2 (8.70)	1 (8.33)	0 (0.00)	--
Parity					
Mean	3.06	3.55	--	--	--
		(*n* = 22)			
Median	2.50	3.00	--	--	--
Range	1–6	1–11	--	--	--
Age of infant (months)					
Mean	6.14	6.45	--	--	--
		(*n* = 22)			
Median	6.50	6.00	--	--	--
Range	0.03–14.00	0.77–13.00	--	--	--
Clinical training					
Clinician^d^	--	--	--	--	3 (25.00)
Non-clinician^e^	--	--	--	--	9 (75.00)

^a^Women who have delivered a child within the preceding 14 months

^b^Partners of postpartum women interviewed

^c^Includes politicians, Muslim clerics and Christian ministers

^d^Includes Clinical Officers, Assistant Clinical Officers and Assistant Medical Officers with the ability to prescribe drugs

^**e**^Includes Enrolled Nurses, Registered Nurses, Nurse Midwives and Nursing Officers who do not prescribe drugs

### Training

Interviews were carried out by six Tanzanian data collectors (three male, three female) with masters-level training in social sciences or public health and with previous experience in qualitative data collection. All data collectors were native *Kiswahili* speakers and they conducted all interviews in *Kiswahili*. The team was accompanied by a researcher from Johns Hopkins University (SAM) who conducted training and supervised field work with the team. The study team received one week of training, which included pilot testing.

### Data collection

Data collection took place between July and September 2011. Upon entering a community, researchers sought guidance from members of village health committees to identify participants fulfilling the study criteria: women who gave birth in the 14 months preceding the study, their partners, public opinion leaders (including religious leaders), and community health workers (CHWs). Consenting participants were interviewed in a setting of their choosing—often their homes or surrounding environs—and IDIs typically lasted 60–90 minutes. In the event that an interview was compromised by the presence of a curious onlooker, the interviewer politely explained the purpose of the interview (“to learn about maternal health”) and requested that the onlooker leave. In two cases, a research manager (SAM) approached especially curious onlookers and engaged them in a separate, informal conversation that was not within the vicinity of the IDI. This approach was effective in maintaining privacy during IDIs.

IDIs focused on knowledge, attitudes and experiences related to careseeking and counseling during the most-recent pregnancy and birth. At the outset of data collection, the research team did not intend to explicitly investigate opinions of and experiences with family planning. However the theme of contraception (coupled with concerns about side effects) emerged in the earliest interviews, and was probed more explicitly as data collection progressed.

### Data analysis

Following each day’s interviews, the field supervisor led debriefing sessions to triangulate findings, strengthen probing among data collectors, identify a need for follow-up interviews and develop themes for a codebook. All interviews were digitally recorded and transcribed by the same data collectors who carried out the interviews. Each transcript was quality controlled by bilingual researchers (JJC and IHM) and coded. Codes were first developed during data collection and later refined via open coding of a representative sample of transcripts. A codebook was developed collaboratively by the data collection team with lead researchers, and codes were applied to all transcripts using ATLAS.ti, a data management tool [24]. Coded texts were then translated into English, coded by the lead author with validation checks by SAM and PJW. In-country debriefings with regional and national stakeholders who included representatives from spheres of academia (Muhimbili University), policy (the MOHSW) and maternal health programming (Jhpiego) further informed the co-authors’ understanding of contextual realities related to contraception, which informed the presentation of this data.

### Ethical clearance

Ethical clearances were obtained from the Institutional Research Boards of Johns Hopkins Bloomberg School of Public Health in Baltimore, USA and Muhimbili University of Health and Allied Sciences in Dar es Salaam, Tanzania and informed consent was provided by all research participants.

## Results

### Respondent characteristics

IDIs were conducted with 100 respondents across five respondent groups (see Table 1): postpartum women [35], their partners [23], community leaders [12], community health workers [19] and facility-based providers [12]. Several follow-up interviews were conducted, but unfortunately this information was not consistently recorded. Two respondents (both women) declined to participate stating that they needed to tend their fields.

Postpartum women participating in this study were aged between 18 and 43 with a median age of 28. Their partners were slightly older, with a median age of 37 (range 23–60). A majority of women (85 %) and their partners (91 %) were married or cohabiting. Half of the women and about two thirds (65 %) of their partners had completed primary school. Reported parity from postpartum women ranged between 1 and 6 children compared to 1 and 11 reported by partners. The median age of the youngest infant, as reported by men and women was 6 and 6.5 months, respectively.

All community leaders were male, most were married (92 %) and had a median age of 42 years (range 29–65). More than half of community leaders (58 %) had completed primary school education and nearly all (92 %) reported the ability to read and write.

CHWs reported high education (68 % completing primary school education) and literacy levels (100 %). CHWs had a median age of 43 (range 25–52) and a majority were married (68 %). Facility- based workers were younger (median age 35) than CHWs, a majority (75 %) were male and were non-clinical nurses (75 %).

### Contraceptive side effects as a pivotal concern

In general, postpartum women participating in this study were conversant with hormonal contraceptive methods and had used them in the past. Women largely held positive views of modern methods of contraception due to their reported health benefits (“It gives my body time to breathe”) and economic benefits (“We can focus more on each child and earn more money”). As one woman explained, “I think [FP] is very good because it reduces the number of people. It is even better because you can increase their level of education [and have a] better life” (32-year-old mother of 2, current condom user). This finding was consistent across age, district and regardless of distance between a woman’s home and the nearest health facility.

When probed regarding attitudes toward contraceptives, a majority of women (25 of 34) reported concerns regarding side effects of hormonal methods, which they consistently described as a major deterrent to use. One woman explained*,* “Every [modern] method seems to have its problems…I have not seen a safe method yet” (20-year-old mother of 1, currently using the calendar method). Another woman who was disillusioned by modern contraceptive methods said, “The calendar [method] is the best because it doesn’t have chemicals or injection” (24-year-old mother of 2, currently using the calendar method).

Whether experienced firsthand by postpartum women or described by other community members, respondents reported that negative side effects frequently led women to avoid future use of hormonal contraceptive methods, discontinue use, switch methods, or use methods incorrectly, which sometimes resulted in unintended pregnancies. As one woman recounted: “I used to get the injection but it was disturbing me- I used to bleed a lot. Therefore, I stopped and started using the pill. After the pill I went back to the injection, stopped again then got pregnant” (39-year-old mother of 5, currently using the injection). Another woman who became pregnant after switching to a less effective FP method said, “My stomach was giving me problems, so I decided to use the calendar [method], but my husband interrupted the calendar” (42-year-old mother of 6, current FP use unknown).

The idea that women sought a series of contraceptive methods “to see which one will match with me” (29-year-old mother of 3, currently not using any method) was expressed by numerous women such as a 20-year-old woman who explained, “If [the implant] affects you, you leave it and use pills” (20-year-old mother of 1, currently abstaining).

The results presented in the following sections outline the types of FP side effects reported by women and their partners, including contaminated breast milk, infertility and sterility, and excessive and prolonged bleeding. We then present the sources of influence that affect women’s decisions to use hormonal FP methods divided by partners, the community, religious leaders and facility-based providers.

### Side effects attributed to hormonal contraceptives

There was some variation in perceived side effects by method (see Table 2), but all hormonal methods were perceived to be associated with contaminated breast milk (*Uchafu*- dirt), infertility/sterility (“… it burns eggs” (24-year-old mother of 2, current FP use unknown)), and excessive bleeding. Women also described stomach pains (“my stomach was on fire” (39-year-old mother of 5, current injection user)), dizziness, fatigue, missed menses and vomiting as side effects of hormonal contraceptives. Among women who have never used hormonal contraceptives, their awareness of negative side effects experienced by others discouraged uptake. For a breakdown of responses to perceived or experienced side effects by respondent type see Table 3.

**Table 2:** Side effects associated with hormonal contraception by respondent group

Commodity	Side effect	Respondent group		
		Mothers	Fathers	Community
Injections/ “Depo”	Bloating	X		
	Missing menses	X		
	Stomach pain	X	X	
	Weight gain	X	X	X
	Headache	X		
Pills	Sterility	X	X	
	Nausea	X		
	Weight loss	X		X
Implants/ “sticks”	Cancer			X
Across hormonal contraceptives	Excessive and/or prolonged bleeding	X	X	X
	Sterility	X	X	X
	Contamination of breastmilk	X	X	X

**Table 3:** Views on hormonal contraceptives and side effects by respondent group

Respondent group	Attitudes toward contraceptives and contraceptive side effects	Response to side effects
Postpartum women	Women are concerned/confused about how hormonal contraceptives function	● Visit health center to speak with provider
	Women link side effects to other illnesses/conditions (e.g., impaired child development, sterility)	● Abstain from, switch or discontinue hormonal contraceptive use
Partners of postpartum women	Husbands/partners primarily concerned about wife/partner experiencing side effects (e.g., losing excessive blood, feeling ill and/or becoming infertile) or passing illness to breastfeeding children.	● Encourage woman to discontinue or change contraceptive method
		● Use condoms to avoid side effects
		● Abstain while partner is breastfeeding
Community members including CHWs, religious and political leaders	Religious authorities pressure families to avoid FP as it “kills God’s eggs” and could extend the duration of menses thereby inhibiting religious participation	● Encourage families to avoid all FP methods—especially hormonal contraceptives
	Community impressions that FP side effects foster laziness and may induce infertility	● Encourage women to discontinue use to avoid fatigue/laziness and infertility
Health care providers	Providers describe challenges to counseling on FP and side effects especially time constraints. Providers have an impression that women are disinterested in counseling and “only want to get the method and go”	● Encourage women to continue with method if side effects are not severe
	Providers perceive the distribution of contraception to be more important than discussion of side effects	● Encourage alternative methods in instance of severe side effects (and provide these methods)
	Providers weigh the benefit of secrecy (associated with injectables) over the drawback of side effects among patients whose husbands oppose contraceptive use	● Allow women to choose their preferred contraceptive method, notwithstanding potential side effects

#### Contaminated breast milk (“My child is too young”)

A common concern expressed by both postpartum women and their partners was a negative impact of hormonal methods on breast milk and, consequently, on their infant’s health. Specifically, respondents said pills spoil breast milk and induce diarrhea, malaise, fever and other life-threatening illnesses among infants. However, women were unable to describe the mechanism by which contraceptives harm their children: “They say if you use [FP] while the child is small, it is affected…now there I don’t understand [how it is affected], I just hear” (29-year-old mother of 3, currently not using any method). Origins of infant health conditions that appeared inexplicable (e.g. an infant who cannot walk) or for which a provider’s explanation was deemed inadequate, were often attributed to hormonal contraception.

In an effort to minimize infant exposure to this perceived contamination (“I do not want to harm my child” (28-year-old mother of 5, currently abstaining)), women often opted to reinitiate the use of hormonal contraceptives once a child passed a developmental milestone: “I would like to use family planning after I see my child is older and can walk, about two years old. Because at this stage, I do not see the use of the injection or pills when my child is still young” (27-year-old mother of 2, currently not using any method).

#### Infertility and sterility

All respondent groups, and health providers in particular, noted the slow uptake of hormonal contraceptive methods in some communities as a result of widespread concerns regarding temporary infertility associated with contraceptive use or, more severely, the onset of sterility. For example, a husband explained: “They say these pills, once they spread in the body; they cause sterility…that’s what community members told us” (38-year-old partner). Sterility was of particular concern among young women, who feared they would have difficulty getting pregnant after prolonged use of pills. Some women attributed sterility to a blocked uterus: “Those pills, when I swallow them, they will go and stay in my womb, one on-top of the other, and then I will not be able to get another child” (28-year-old mother of 5, current FP use unknown). Missed menses and amenorrhea were seen as proof of sterility, and therefore were a concern for women: “You can stay up to three months like a man [without menstruation]” (30-year-old mother of 3, current condom user). A slightly older woman added, “And those injections, how long will I use them? At the end you will have to stop using [them]. There are women who are advised that you can use these injections continuously and later, you are unable to give birth” (39-year-old mother of 5, current injectable user).

#### Excessive and prolonged bleeding

Women described how long-term bleeding associated with all hormonal contraceptives, and injections in particular, interrupted their day-to-day lives. Women noted that bleeding impeded their ability to perform domestic duties, tend to their families and earn a living. Some women also pointed out that prolonged bleeding interrupted their religious practice, since women can be prohibited from handling religious books while menstruating: “When you use the injection, you get heavy and experience long [menstrual] bleeding. Therefore, you are not able to pray…you cannot hold the book of God until the bleeding stops” (35-year-old mother of 4, currently not using any method).

### Sources of influence regarding family planning use

#### Partner

A few women interviewed disclosed that their partners, or other men they know, discourage and/or oppose the decisions of their wives to use family planning. As one woman explained, “They [male partners] refuse. They really don’t like us using family planning” (18-year-old mother of 1, not currently using any FP method). Men who agreed with this statement most frequently cited adverse side effects experienced by their wives as their primary reason for opposition.

Partners of postpartum women were generally responsive to physical distress their partners experienced from using hormonal contraceptives: “[My husband] is afraid of the problems that I might get” (18-year-old mother of 1, not currently using any FP method). To avoid negative side effects, women and husbands reported using condoms or switching to traditional methods (rhythm or calendar methods). While switching to condoms was undesirable for some men (using condoms is like “licking sugar with the wrapper still on…you cannot taste the sweetness” (A male CHW)), some husbands were willing to use condoms to avert the side effects of hormonal methods: *“*When I saw that the injection caused her to have stomach problems, she stopped and started using the pill. When I saw that the pill was also giving her problems, I made her stop and I started using condoms” (34-year-old partner).

Women and men also reported abstaining from sex after childbirth, a time period that ranged from 40 days to 5 years, but typically lasted 2 years. Partners of postpartum women were forceful in agreeing that women should not take hormonal methods while breastfeeding: “My wife is still breastfeeding, how can you use [contraceptives] while she is breastfeeding?” (42-year-old partner).

#### Community

The community itself was shown to play an important role in disseminating views on FP side effects. Women reported being influenced by the views of community members. One woman described how older community members scorn families using contraceptives: “At home, I used to hear now and again, even older women used to say, 'You use contraceptives? Contraceptives are bad! They will just hurt you'” (30-year-old mother of 5, current injection user). However, negative views of hormonal methods were not universal, as one woman explained: “The community cannot discriminate against her, when she gets side effects, she stops. The community does not discriminate because it is a personal decision” (24-year-old mother of 2, currently using the calendar method).

A commonly held perception across community members was that hormonal methods induce side effects such as excessive bleeding, which leads to laziness. This perception caused community members to gossip about suspected FP users, thereby discouraging uptake. A husband explained: *“*Some will say she is using family planning because she does not like kids, some will say she is lazy and she does not like to farm, everyone will say his or her own things. This harassment will lead others not to use family planning methods*”* (38-year-old partner).

#### Religious leaders

According to all respondent groups, religious leaders’ views also affect contraceptive uptake. Interviewed religious leaders objected to FP as it “wasted” or “killed” sperm or eggs. One Muslim religious leader lamented that “year after year” husbands and wives using contraceptives are preventing birth: “Contraceptives kill God’s eggs.” Only one of seven interviewed religious leaders, a Christian pastor, expressed positive sentiments towards the use of contraceptives, saying, “I give advice about family planning, because God gave us [sexual] urge.” Although there was opposition to the use of contraception from both Christian and Islamic groups, leaders from both religions showed leniency toward married couples using contraceptives. However, many families have internalized disapproval of religious leaders: “My religion does not allow me [to use contraceptives]” (35-year-old mother of 4, currently not using any method). Another woman continued to say, “They [religious leaders] disagree about FP. They want us to have children. They don’t agree. We kill eggs when we use FP” (24-year-old mother of 4, currently using pills).

#### Facility-based providers

Most women reported that health care providers did not counsel them about side effects that can be expected for particular contraceptive methods: “[A provider] comes in and tells you that you should use FP if you want good health…they tell you to use this injection, but they do not tell you the side effects” (42-year-old mother of 6, current FP use unknown). Another woman expressed frustration, saying, “No one educates us! You are just told [to use FP] until the nurse gets angry [and says], ‘You come and tie your tubes, you have given birth to enough children, it’s enough now!’”(39-year-old mother of 5, current injectable user). One woman described how an absence of facility-based counselling fosters distrust and leads women to bypass providers for informal pharmacies where they self-prescribe inappropriate methods: “They use medication that does not suit them. For example, if you have high blood pressure, you have to go and get examined; they look at your uterus. If you have high blood pressure, pills are not for you. What suits you is the injection or an implant. Now, they take a method themselves without undergoing tests” (28-year-old mother of 5, currently not using any method).

However, some women expressed a more cordial relationship with their service providers. One woman said, “They [facility-based health providers] told us, if you see differences—that one [method] affects you, go there [to the facility]. If you go there they test you, then they change [the method] for you” (39-year-old mother of 6, current injection user).

Providers emphasized that their aim is to encourage FP uptake and meet demand for FP within time and supply constraints. While providers could detail side effects in interviews, they noted that women often come to facilities with a particular method already in mind and are determined to use that method, which makes discussions about side effects challenging: “They choose what they want. If they want Depo [likely referring to the injectable progestogen-only brand of hormonal contraceptive Depo-Provera], we give them Depo. If they want pills, we give them pills. If they want implants, we give them implants. What they want is what we give them” (Provider, Enrolled Nurse).

Providers encouraged method switching in response to severe side effects, but urged women to “be patient” and wait for less severe side effects to pass: “You advise them that this is how these medications are. If you are not very ill and you are just losing weight that is not a problem. But if you have headaches or are bleeding a lot, we have to change [contraceptive] methods” (Provider, Nurse Midwife).

Providers also described how the benefit of secrecy outweighs the drawback of side effects among many patients. Several providers described how women appreciate injections because this method is long-lasting yet discreet, despite protracted bleeding: “Their husbands do not like it [FP] at all. We tell them to bring their husbands so that we can counsel both of them and they refuse. They tell you, ‘Nurse, it is not easy for me to take pills. Give me the injection.’” (Provider, Nurse Midwife).

## Discussion

Our study adds qualitative context to a largely quantitative body of research. Through triangulated data across key respondent groups, we have highlighted that women refrain from reinitiating FP post-delivery largely out of concern that contraceptives may negatively affect their health and the quality of their breast milk. Other contributors to discontinuation and delayed reintroduction are linked to misinformation from community members about infertility, religious opposition, and community perceptions of FP as fostering laziness or inducing fatigue. Nationally representative surveys highlight that adverse side effects are a leading reason for contraceptive discontinuation reported by Tanzanian women (22.5 %), second only to a desire to become pregnant (37.5 %) [25]. Addressing concerns about side effects is therefore critical to achieving the Tanzanian government’s goal of increasing modern contraceptive prevalence [26].

Over the past several decades, studies have described how a woman’s firsthand experience or secondhand knowledge of adverse side effects may present critical constraints to family planning uptake or use [4–9]. Although there are clinically known side effects associated with hormonal contraceptives—including, from the estrogen component, nausea, headaches, and breast cancer and, from the progestin component, weight gain, depression, fatigue and irregular menstrual bleeding—serious side effects are generally rare and contraceptives are generally regarded as safe [27]. Nevertheless, studies have shown that perceived and experienced side effects of hormonal contraceptives influence perceptions of modern contraception and, consequently affect women’s decisions to begin, change, or continue FP use [28–36]. A systematic review of qualitative research across low-income settings—not specific to the PPP—found that menstrual disruption and a risk of infertility were identified as the most influential factors considered by women when deciding to avoid or discontinue use of hormonal contraception [37].

An important part of ensuring contraceptive continuation is providing high quality FP services, which should include counseling on the effective management of side effects. Such counseling could be provided across the maternal and newborn health continuum of care (at interactions that occur during the antenatal, delivery and postpartum periods). Unfortunately, women interviewed in this study reported receiving little or no FP counseling prior to being prescribed an FP method. Women also described how they felt uninformed of possible side effects or lacked guidance on how to respond to them. These findings are consistent with DHS data, which have found that many Tanzanian women—50.4 % of pill users, 43.9 % of injectable users, and 27.1 % of implant users—report that they were not counseled on side effects upon FP uptake [13]. This lack of information affects how women respond to the experience of side effects and can also underscore a family’s decision to bypass health facilities or to place their trust in inaccurate information circulating in their communities. Therefore, we urge that programs ensure high quality FP/PPFP counseling, and that providers place pointed emphasis on deep-seated concerns expressed across respondent groups involving sterility and poor infant health.

Literature related to PPFP often describes the PPP as an ideal time to promote counseling on (and adoption of) FP because women are assumed to be making frequent contact with the health system. In Tanzania, frequent interaction is not occurring. At present, just half of women deliver in a facility [13]. In the 42 days following birth, 65 % of women do not return to health facilities for a postpartum visit [13]. We posit that this lack of interaction with the health system undercuts women and their partners’ ability to draw informed understandings about FP. Amid efforts to promote more engagement between women, their partners and facility-based health providers, we echo existing literature that argues in favor of bringing health messages to communities via community health workers and we also encourage more discussions of FP during antenatal visits and prior to discharge (in the event of facility-based births) [5, 38]. A series of interventions that program managers could adopt to address PPFP has been outlined in Gaffield 2014.

Unfortunately, to date the evidence of what works in fostering PPFP is “very low,” according to a 2014 Cochrane Review [39]. This dearth of evidence underscores a need for partnerships across research and intervention groups in order to identify meaningful, effective, and culturally appropriate mechanisms to address this problem.

### Study limitations and opportunities for future research

The nature of rapid qualitative data collection may have resulted in limitations including interviewer and informant fatigue and social desirability bias. It is valuable to highlight that this study offers insight into a range of experiences and attitudes across several information-rich respondent groups, but does not assess the proportion of each population that would report these. Additional quantitative research could shed light on rates of reported side effects and proportions of the population that hold specific attitudes related to contraception and side effects.

## Conclusions

Perceived and experienced side effects resulting from the use of hormonal methods of contraception can discourage uptake and encourage discontinuation of these methods. Our study emphasizes a need for health providers to expand the amount of information relayed to women, their partners and community leaders on the topic of contraception, including both the benefits of family planning and the reality of contraceptive side effects. We urge that representatives of the health system (whether based in facilities or communities) counsel women on how to choose an FP method, outline the side effects associated wtih various methods, and provide support in the event that side effects need to be mitigated. We also urge that providers address community concerns regarding a link between contraception and sterility or impaired child development.

## Abbreviations

CHW: Community Health Worker
CPR: Contraceptive Prevalence Rate
FP: Family Planning
IDI: In-Depth Interview
LAM: Lactational Amenorrhea Method
MOHSW: Ministry of Health and Social Welfare
PPFP: Postpartum Family Planning
PPP: Postpartum Period
SSA: Sub-Saharan Africa
TFR: Total Fertility Rate

## Footnotes

1 At the time of data collection, Kilosa District Council (DC) was one district. Kilosa DC was later divided into two districts: Gairo and Kilosa DC.
